# Patellofemoral Pain in Elite Female Volleyball Players: Correlation With Trunk Muscle Strength

**DOI:** 10.7759/cureus.88729

**Published:** 2025-07-25

**Authors:** Dimitrios Kalatzis, Georgios Zoumpoulis, Konstantinos Zygogiannis, Konstantinos Kaoullas, Ioannis Fotoniatas, Georgios C Thivaios

**Affiliations:** 1 Orthopedics and Traumatology, Laiko General Hospital of Athens, Athens, GRC; 2 Orthopedics - Scoliosis and Spine, KAT General Hospital, Athens, GRC

**Keywords:** adolescent athletes, isokinetic dynamometry, patellofemoral pain, trunk muscle strength, volleyball

## Abstract

Background

Patellofemoral pain (PFP) is a prevalent musculoskeletal condition, particularly among adolescent and young adult females, often associated with sports involving high knee loads, such as volleyball. While previous studies have examined local and distal factors contributing to PFP, the role of trunk muscle strength remains underexplored, particularly in elite athletes.

Objective

The objective of this study is to investigate the correlation between PFP and trunk muscle strength in elite adolescent female volleyball players.

Methods

A retrospective analysis was conducted on 23 elite adolescent female volleyball players (mean age: 15.59 years) from the Hellenic National Team. Trunk muscle strength was measured using isokinetic dynamometry (Biodex System 4 Pro, Biodex Medical Systems, Inc., Shirley, NY, USA) during maximum flexion and extension at 90° of trunk inclination. Participants were categorized into PFP (n = 11) and non-PFP groups (n = 12) based on clinical evaluation. Statistical analysis included comparison of peak torque (Nm/kg), mean power, and agonist-antagonist ratios between groups.

Results

No significant differences were found in trunk flexion strength (p = 0.449), trunk extension strength (p = 0.423), or agonist-antagonist ratios (p = 0.601) between PFP and non-PFP groups. Trunk extension strength was consistently greater than flexion strength across all participants. Most athletes with PFP reported pain onset linked to increased training loads.

Conclusions

Despite the hypothesized correlation, this study did not identify significant differences in trunk muscle strength between volleyball players with and without PFP. These findings contrast with prior research on non-athletic populations, suggesting that elite athletes may possess compensatory mechanisms. Further studies are warranted to clarify the role of trunk strength in PFP pathophysiology and inform targeted prevention and rehabilitation strategies in elite sports.

## Introduction

Patellofemoral pain (PFP) is a common musculoskeletal disorder, with an annual prevalence of 22.7% in the general population and 28.9% among adolescents [1]. PFP is defined as pain around or behind the patella, which is aggravated by at least one activity that loads the patellofemoral joint during weight bearing on a flexed knee (e.g., squatting, stair ambulation, jogging/running, and hopping/jumping) [2]. PFP is more common in females. Specifically, its incidence among adolescent females and young adult women has been reported to be two to 10 times higher than in their male counterparts [3]. Moreover, early sport specialization is associated with an increased risk of developing PFP [4,5]. PFP is one of the most frequent overuse injuries among volleyball players [3,6,7].

Anterior knee pain, the main symptom of PFP, can limit an athlete’s performance or even lead to sport cessation in the recreational athlete, highlighting the high impact the syndrome has on the quality of life [4]. Earlier hypotheses supported that PFP has a self-limiting course. On the contrary, current literature suggests that PFP is a refractory knee condition. PFP persists in 50% of individuals and in some cases for as long as 20 years [5].

PFP is a common clinical complaint, particularly among active individuals, and requires careful differential diagnosis to exclude other potential sources of anterior knee pain. Conditions that may mimic PFP include Osgood-Schlatter disease, particularly in adolescents, and plica syndrome, which may present with similar anterior knee discomfort and mechanical symptoms. Overuse injuries, such as patellar tendinopathy and quadriceps tendon tendinopathy, can cause localized pain that overlaps with PFP in both location and aggravating activities. Patellar instability, including subluxation or dislocation, must also be ruled out, especially in patients with a history of trauma or a sensation of giving way. Meniscal tears, particularly of the anterior horn, and prepatellar bursitis may also contribute to anterior knee pain and should be differentiated through clinical examination and imaging if necessary. Additionally, referred pain from the hip should be considered. Finally, chondromalacia patellae, characterized by softening and degeneration of the patellar cartilage, is a structural condition that may underlie and can be identified with advanced imaging. A comprehensive clinical assessment is essential to accurately diagnose PFP and guide appropriate management [8-11].

The etiology of the syndrome is complex. Many factors, including anatomical, biomechanical, and psychological, contribute to the development of the pain [12]. The majority of young patients exhibit no structural damage in the patellofemoral joint [4]. The latest pathomechanical model analyzing PFP syndrome supports that elevated joint stress (abnormal loading of the patellofemoral joint) is the key factor [12]. A variety of imbalances, both local (within the knee joint) and distal (in the hip and trunk), can contribute to abnormal loading patterns [12]. Numerous studies have investigated local factors such as impaired quadriceps function, reduced patellar cartilage, and impaired soft tissue restraints. However, few studies have examined the relationship between trunk muscle performance and function and PFP. Therefore, current evidence is insufficient to determine with certainty whether individuals with PFP exhibit trunk muscle impairments. To address this gap, we assessed trunk muscle strength in 23 elite adolescent female volleyball players, both with and without PFP, to test the hypothesis that those with PFP would demonstrate reduced trunk muscle performance.

## Materials and methods

We retrospectively analyzed data from 23 elite volleyball players. All participants were female, aged between 14 and 17 years old (mean age: 15.59). These athletes were all members of the Hellenic Volleyball National Team. Trunk strength was assessed using the isokinetic Biodex System 4 Pro (Biodex Medical Systems, Inc., Shirley, NY, USA). Isokinetic dynamometry has been one of the most widely used approaches to assess the strength of trunk muscles due to its high reliability and precision [13]. Measurements of peak torque per body weight and mean power of flexion and extension of the trunk at a stable angle of 90 degrees were made.

Subjects were tested in a seated position with the anterior iliac spines in alignment with the fixed axis of rotation of the Biodex unit. Stabilization straps were placed across each subject, applied over both hips, on the upper trunk, and on the proximal thighs. A 10-minute treadmill warm-up and a familiarization trial were performed in order to help athletes acclimate to the equipment. Afterward, the participants performed maximum isokinetic trunk flexion and extension. Verbal encouragement was provided throughout the entire testing procedure to ensure maximum effort. Participants who exhibited signs and symptoms indicative of the following conditions, other than PFP knee pathology, were excluded from the study: (1) patellar tendinopathy; (2) history of knee surgery; (3) meniscal injury; (4) collateral ligament injury; (5) patellar instability; and (6) referred pain from the spine or hip. Examination was performed by the same orthopedic surgeon. We initially enrolled 23 athletes, none of whom met the exclusion criteria, and thus all 23 participants were included in the final analysis. Outcome assessors were blinded to the participants’ PFP status to minimize potential bias during strength testing.

For continuous variables, mean, SD, and range or median, 25th and 75th percentiles, and range were calculated after testing for normal distribution. For categorical variables, frequencies and percentages were calculated. The continuous variables were tested for normality using a Shapiro-Wilk test. Mann-Whitney U tests and unpaired t-tests were applied for the comparison of the continuous variables depending on the distributions. For the graphical presentation of the data, box plots were created. All statistical analyses were performed using Stata/IC version 15.1 (StataCorp LLC, College Station, TX, USA), and the significance level was a = 0.05.

## Results

A total of 23 participants were included in the study, of whom eleven (47.83%) reported PFP. Among these, 10 reported unilateral pain, while one reported bilateral symptoms. The vast majority of athletes presenting with PFP correlated the development of their pain with excessive training load. We also evaluated selected anthropometric characteristics of the participants (age, height, weight, and BMI) (Table 1).

**Table 1:** Anthropometric characteristics of the athletes

Parameter	Mean	SD	Min	Max
Age (years)	15.59	0.6	14.18	16.82
Height (cm)	176.94	8.86	154.5	187
Weight (kg)	68.86	9.58	48.6	91.1
BMI (kg/m²)	21.91	1.8	19.4	26.9

Trunk flexion and extension strength, normalized to body weight, were measured at a fixed angle of 90 degrees. Participants with PFP did not report any pain during isokinetic dynamometry testing. No significant differences were observed in trunk flexion strength (p = 0.449) or trunk extension strength (p = 0.423) between participants with and without PFP (Table 2, Table 3). Additionally, there were no significant differences in the agonist-antagonist ratio between the two groups (p = 0.601) (Table 4).

**Table 2:** Trunk flexion per body weight PFP, patellofemoral pain

Flexion Tq peak (Nm/kg)	Mean	SD	Range
Population	1.726	0.358	1.13-2.37
PFP	1.786	0.355	1.13-2.28
No PFP	1.67	0.367	1.19-2.37

**Table 3:** Trunk extension per body weight PFP, patellofemoral pain

Extension Tq peak (Nm/kg)	Median	25th-75th percentile	Range
Population	3.3	2.37-3.66	1.23-3.96
PFP	3.4	2.37-3.66	1.6-3.96
No PFP	3.145	2.335-3.635	1.23-3.96

**Table 4:** Agonist-antagonist ratio PFP, patellofemoral pain

Agonist-antagonist ratio	Median	25th-75th percentile	Range
Population	52.1	45.9-64.6	34.7-109.2
PFP	52.1	46.8-64.6	43.6-109.2
No PFP	50.8	43.7-62.75	34.7-72.9

## Discussion

In the literature, considerable emphasis has been placed on analyzing local factors contributing to the development of PFP. Numerous studies have also investigated distal factors, including hip muscle function, as well as foot and ankle strength, structure, and mobility. However, the number of studies examining trunk muscle strength and function remains notably limited.

dos Reis et al. [14] suggested that individuals with PFP exhibit differences in trunk kinematics compared to pain-free controls. Specifically, they demonstrated altered trunk kinematics in the frontal plane, characterized by an ipsilateral trunk lean during single-limb tasks, such as jumping and landing. This compensatory movement may shift the body’s center of mass toward the stance limb, thereby increasing the knee abductor internal moment and the patellofemoral joint stress.

Cowan et al. [15] demonstrated altered hip and trunk muscle function in individuals with PFP. Specifically, they identified reduced trunk side flexion strength associated with PFP, based on electromyographic activity recorded using surface electrodes.

Briani et al. [16] investigated the thickness of obliquus internus (OI), obliquus externus (OE), transversus abdominis, and rectus abdominis muscles in two groups of women: a PFP group and a pain-free group. Muscle thickness was assessed using B-mode ultrasound imaging. Their findings revealed reduced thickness of the OE and OI muscles in women with PFP.

Manojlović et al. [17] investigated trunk strength, flexibility, and postural control in individuals with unilateral or bilateral PFP compared to asymptomatic controls, using custom-made dynamometers. Their results indicated trunk muscle deficits in participants with PFP relative to those without the condition.

Trunk muscle strength plays a crucial role in both health and physical performance, as it is closely linked to trunk and lower limb stability. High-level athletes have been shown to possess greater trunk extension strength relative to flexion strength compared to non-athletic individuals [18]. In healthy, non-athletic populations, the typical trunk flexion-to-extension strength ratio ranges from 0.7 to 0.9, whereas in athletes, it tends to be lower, ranging between 0.5 and 0.7 [18,19]. The majority of athletes in our study (20 out of 23; 86.95%) demonstrated a trunk flexion-to-extension strength ratio of less than 0.7, while only three athletes (13.05%) exhibited a ratio greater than 0.7.

To our knowledge, no study has investigated trunk muscle strength in individuals with PFP using the Biodex isokinetic dynamometer. Moreover, no research has specifically examined the relationship between PFP and trunk muscle strength in female volleyball players, a population with a high incidence of PFP. Therefore, we assessed trunk muscle strength in 23 adolescent female volleyball athletes, with and without PFP symptoms, using the Biodex dynamometer. Based on existing literature, our initial hypothesis was that participants with PFP would demonstrate reduced trunk muscle strength compared to their pain-free counterparts. However, our results did not reveal any statistically significant differences in trunk muscle strength between athletes with and without PFP. This finding contrasts with previous research, as discussed earlier. Given the limited number of studies on this topic and the conflicting evidence to date, further research is warranted to clarify the relationship between trunk muscle strength and PFP.

This study has several limitations that should be acknowledged. First, the small sample size (n = 23) limits the statistical power and generalizability of the findings. While the cohort represents elite adolescent female volleyball players - a highly specific and relevant population - the results may not be applicable to broader athletic or general populations. Second, the retrospective design inherently carries a risk of bias, particularly recall bias, as participants may have had difficulty accurately reporting the onset or history of PFP. Third, the cross-sectional nature of the study precludes any inference of causality between trunk muscle strength and the presence of PFP. Finally, while the isokinetic dynamometer provides reliable strength measurements, it does not capture dynamic trunk control or endurance, which may also contribute to patellofemoral mechanics.

## Conclusions

Female volleyball players exhibit a high incidence of PFP, which can negatively impact both athletic performance and quality of life. The pathophysiology and pathomechanics of PFP in this population are complex and remain insufficiently documented. In our experimental study, no statistically significant trunk muscle strength imbalances were identified in athletes with PFP. Further research is necessary to determine whether trunk muscle strength constitutes a predisposing factor for PFP in elite athletes. Such evidence would inform the development of targeted preventive exercise programs for individuals with trunk muscle impairments and support more effective rehabilitation strategies when PFP is already present. Therefore, a deeper understanding of the interaction between trunk function and lower extremity kinematics is essential in optimizing the management of professional athletes.
